# Tailoring a national smoking cessation support programme in co-creation with (expectant) parents in vulnerable situations

**DOI:** 10.1186/s12889-026-26633-9

**Published:** 2026-02-14

**Authors:** Linda van der Spek, Anna L. de Bree, Tessa Scheffers-van Schayck, Hans van Kippersluis, Hilmar H. Bijma, Gera E. Nagelhout, Jasper V. Been, Leonieke J. Breunis

**Affiliations:** 1 Division of Neonatology, Department of Neonatal and Paediatric Intensive Care, Erasmus MC Sophia Children’s Hospital, University Medical Centre Rotterdam, Dr. Molewaterplein 40, Rotterdam, The Netherlands; 2 Department of Epidemiology, Trimbos Institute, Da Costakade 45, Utrecht, The Netherlands; 3 Department of Youth, Trimbos Institute, Da Costakade 45, Utrecht, The Netherlands; 4 Erasmus School of Economics, Erasmus University Rotterdam, Burgemeester Oudlaan 50, Rotterdam, The Netherlands; 5 Division of Obstetrics and Fetal Medicine, Department of Obstetrics and Gynaecology, Erasmus MC Sophia Children’s Hospital, University Medical Centre Rotterdam, Dr. Molewaterplein 40, Rotterdam, The Netherlands; 6 Department of Care Ethics, University of Humanistic Studies, Kromme Nieuwegracht 29, Utrecht, The Netherlands; 7 Department of Health Promotion (CAPHRI), Maastricht University, Universiteitssingel 40, Maastricht, The Netherlands; 8 Centre of Expertise Perspective in Health, Avans University of Applied Sciences, Lovensdijkstraat 63, Breda, The Netherlands; 9 Department of Obstetrics and Gynaecology, Erasmus MC Sophia Children’s Hospital, University Medical Centre Rotterdam, Dr. Molewaterplein 40, Rotterdam, The Netherlands; 10 Department of Paediatrics, Erasmus MC Sophia Children’s Hospital, University Medical Centre Rotterdam, Dr. Molewaterplein 40, Rotterdam, The Netherlands

**Keywords:** Smoking Cessation, Co-creation, Parents, Pregnancy, Vulnerable Populations, Health Inequities

## Abstract

**Background:**

Smoking prevalence and limited access to cessation support contribute to persistent health inequities among (expectant) parents in vulnerable situations. Co-creation may help align smoking cessation support with the lived contexts and needs of this group. This study documents and evaluates the use of co-creation to explore contextual barriers and needs, and to inform adaptations to *Smoke-free Parents* (SFP), a Dutch telephone-based smoking cessation programme.

**Methods:**

Five co-creation sessions were conducted with (expectant) parents, smoking cessation counsellors, healthcare professionals, and researchers. Activities included photo-based brainstorming, peer interviews, creating fictional user profiles, and user journey mapping. Participants identified barriers to engagement with SFP and retention in SFP, brainstormed adaptations, and prioritized these. Data were thematically analysed. The co-creation process and its outputs were evaluated through interviews and a group discussion.

**Results:**

Three parents, two counsellors, two healthcare professionals, and two researchers participated. Identified contextual barriers included stressful life circumstances, pro-smoking social norms, shame, fear of failure, and limited discussion of smoking and cessation support with professionals. Participants highlighted the need for accessible and personalized services and involving social networks. Proposed adaptations included raising awareness of SFP among professionals and (expectant) parents, offering more diverse forms of coaching (telephone, video call, group, face-to-face), and facilitating involvement of partners or other supportive persons. Participants evaluated both the co-creation process and outcomes positively (mean satisfaction score 8.3/10).

**Conclusions:**

This study illustrates how co-creation can be used to explore context and needs related to smoking cessation support among (expectant) parents in vulnerable situations, and to inform adaptations. Wider use of such co-creation approaches may support more equitable access to cessation support.

**Supplementary Information:**

The online version contains supplementary material available at 10.1186/s12889-026-26633-9.

## Key messages

**What is already known on this topic:**

Socioeconomic disadvantage limits both access to and effectiveness of smoking cessation support, yet few programmes involve parents in vulnerable situations in their development, despite this being key to reducing inequalities.

**What this study adds:**

This study shows how co-creation with parents, counsellors, and professionals can identify real-world contextual barriers to engaging with support and generate concrete adaptations to improve accessibility, personalisation, and social support.

**How this study might affect research, practice or policy:**

These findings offer direction for policymakers and professionals on how systematic use of co-creation in smoking cessation programme development can improve reach, relevance, and equity, supporting efforts to reduce socioeconomic inequities in tobacco-related harm.

**Supplementary Information:**

The online version contains supplementary material available at 10.1186/s12889-026-26633-9.

## Introduction

Smoking is a major contributor to health inequities that emerge early in life, particularly through differences in smoking prevalence and cessation outcomes between population groups [1, 2]. Higher smoking prevalence among (expectant) parents facing social and economic disadvantage contributes to greater risks of serious adverse health outcomes for themselves and their children [3–6]. In the Netherlands, these inequities are clearly reflected in educational gradients in smoking during pregnancy, with substantially higher prevalence among women with lower educational levels (20%) compared to those with higher educational levels (3%) [7]. Modelling studies suggest that eliminating smoking could reduce inequities in stillbirths and infant deaths by up to 40%, highlighting smoking cessation as a key leverage point for reducing early-life health inequities [8].

The inequities in smoking prevalence and cessation outcomes are driven by increased vulnerability arising from an imbalance between risk factors and protective resources [9], with vulnerability understood here as shaped by people’s social and economic situations rather than inherent characteristics of individuals or groups [10, 11]. In the context of smoking cessation, factors such as unstable housing, finances, or relationships, poor mental health, stress, stigma, comorbidities, and pro-smoking norms contribute to higher smoking prevalence and reduced capacity to quit [12, 13], while protective factors like social support and adequate services are often lacking [12, 14]. Smoking cessation support is often less accessible and less well-suited to those who need it most [12, 14–18], consistent with the Inverse Care Law [19]. Many existing programmes are developed based on generalized behavioural theories and evidence from more advantaged groups, which risks limiting their accessibility and perceived relevance for people in more vulnerable situations [12]. There is growing recognition that aligning interventions more closely with the lived context, needs, and preferences of service users may help improve their accessibility and relevance, particularly for groups facing vulnerability [15, 17, 20–23].

Co-creation is one approach that seeks to operationalize this alignment by bringing together academic and non-academic stakeholders, such as service users, healthcare professionals, and counsellors, to jointly design and adapt interventions [24, 25]. Evidence from other areas of public health suggests that co-creative approaches can increase access and outcomes [26]. To the best of our knowledge, co-creation has not yet been used to adapt smoking cessation support together with (expectant) parents in vulnerable situations. In the remainder of this paper, the term ‘parents’ is used to refer to individuals who are planning to conceive, are pregnant, or are parenting young children, including their partners. Existing studies on smoking cessation support in this population have predominantly focused on evaluating intervention effectiveness [27–30] or identifying barriers to cessation [12, 31] as separate lines of inquiry, with limited attention to how service users and professionals can jointly explore their contexts and needs and inform the adaptation of (established) programmes. As a result of the gap in documentation and evaluation of co-creation endeavours, important opportunities to improve not only effectiveness but also reach and sustained engagement may remain underexplored, particularly for those most affected by smoking-related inequities and as (expectant) parents, a critical life stage for the emergence of health inequities.

In this study, we therefore used a co-creation approach to inform adaptations to Smoke-Free Parents (SFP), a national telephone-based smoking cessation programme in the Netherlands [32]. Although the programme has successfully reached many parents through referrals by healthcare professionals or online self-registration, participation could be expanded further. According to healthcare professionals, major barriers to referral include hesitation to discuss smoking and cessation support, along with perceived parental resistance to smoking cessation assistance [33]. In 2024, 739 parents were signed up for SFP, of whom 52% started the programme [34]. Among those who did not participate, counsellors were unable to reach approximately one-third despite prior consent to be contacted, and another third did not want to quit smoking or receive telephone counselling support [35].

Specifically, this study aimed to: (1) explore the contexts and lived experiences of parents, smoking cessation counsellors, and healthcare professionals, as well as their needs and preferences regarding smoking cessation support for parents in vulnerable situations; (2) jointly propose adaptations to SFP to improve its accessibility and relevance for this population; and (3) evaluate the co-creation process itself in terms of participants’ satisfaction, ownership, validity, and perceived added value.

## Methods

This study was reported in line with the Standards for Reporting Qualitative Research [36]. The methods section outlines the researcher characteristics and reflexivity, study context, participants, ethics, the study design, co-creation process, and the evaluation approach.

### Researcher characteristics and reflexivity

The researchers both facilitated and participated in the co-creation process, aiming to minimize power imbalances and engage as much as possible as equal partners. The researchers worked as postdoctoral researcher and paediatrician in training, and as a graduate public health researcher. Across the research team, prior smoking experience ranged from regular smoking to occasional or no smoking. Neither researcher was in a vulnerable situation nor an (expectant) parent. Their perspectives may therefore have differed from those of participants with lived experience of parenthood and vulnerability. To support balanced interpretation, researchers and other participants jointly reflected on the findings during and after the co-creation process, integrating complementary insights from different roles and experiences.

### Study context

For the purposes of this study, the following section provides the necessary contextual information to understand the evaluated Smoke-Free Parents (SFP) programme. SFP is a national smoking cessation programme offering at least six structured telephone counselling sessions based on Motivational Interviewing, complemented by a brochure with information and tips, and delivered by trained cessation counsellors. The programme is supported by evidence from randomised controlled trials demonstrating its effectiveness [32, 37], and studies evaluating its implementation and recruitment approaches [33, 35].

Since its nationwide scale-up in 2019, SFP has been accessible through referrals by healthcare professionals (e.g., youth health services, fertility care, and obstetric care) and through self-registration via the national smoking cessation website www.ikstopnu.nl (promoted on cigarette packages). The programme, which is reimbursed by Dutch health insurers without deductible costs, was gradually expanded to include pregnant individuals and their partners (2020), and people at the preconception stage (2021) [32–35, 38].

Regarding the broader context, in 2018 the Dutch government and over 70 societal, corporate, and healthcare organisations signed the National Prevention Agreement, which aims for a smoke-free society (< 5% smoking prevalence and zero smoking during pregnancy) by 2040. Complementary initiatives such as the Smoke-free Generation campaign and the Taskforce Smoke-free Start seek to raise awareness, urgency, and competence among professionals supporting parents who smoke [38, 39]. Despite these efforts, fewer than half of pregnant women who smoke recall discussing smoking with a healthcare professional [7].

### Sampling strategy

The aim was to recruit 12 to 14 co-creators, as recommended by the Co-creation Framework [24]. Co-creators included Dutch-speaking adults from four stakeholder groups: 1) Smoking cessation counsellors (i.e., service providers) with a minimum of two years of experience in delivering the SFP programme; 2) Healthcare professionals (i.e., referrers) with at least two years of experience in providing care to parents, preferably representing obstetrics, paediatrics, and youth healthcare; 3) Researchers; and 4) parents (i.e., (potential) service users) living in vulnerable situations who smoke tobacco cigarettes or have recently quit smoking tobacco cigarettes. The latter group included individuals planning to conceive, pregnant women and their partners, and parents of children aged 0–18 years. Vulnerability was defined by the researchers as having a low educational level (International Standard Classification of Education levels 0–2 [40]), being unemployed, and/or reporting insufficient income to meet basic needs. In addition, participants faced at least one other vulnerability factor, such as limited social support, chronic or psychiatric illness, or financial distress [9]. These characteristics were determined based on self-report. At inclusion, eligible parents were smoking at least one cigarette per day or had quit within the past three months.

Healthcare professionals were recruited via existing networks (e.g., Taskforce Smokefree Start ambassadors) and local organizations in the Utrecht region, while SFP programme counsellors were invited directly. Professionals and counsellors received and returned an informed consent form by mail. parents were recruited via smoking cessation counsellors, healthcare professionals, community care teams, and social media advertisements. As recruitment involved open calls and snowball sampling, the total number of individuals approached could not be specified. Among those who expressed initial interest but did not participate, the main reasons for non-participation were lack of time, travel distance, and competing personal circumstances, reflecting the substantial demands of study participation, which involved five in-person evening sessions.

Initial expressions of interest were followed by an explanatory text message and phone call from the coordinating investigator (LS), along with an information letter, study brochure, and informed consent form sent by mail. Snowball sampling was used as a secondary strategy, allowing participants to refer others and share a recruitment flyer [41, 42]. After verbal consent, parents were visited at a location of their choice by the coordinating investigator (LS) for an in-person introduction, written informed consent, and a short informal intake interview.

### Participant characteristics

The co-creation group consisted of three parents who smoked or had recently quit, two smoking cessation counsellors, two healthcare professionals (one youth health care nurse specialist, and one midwife), and two researchers. Table 1 provides an overview of participant characteristics. All participants self-identified as women and ranged in age from 27 to 55 years. Healthcare professionals and counsellors were all non-smokers. Parents varied in smoking history and motivation to quit. One signed up for SFP and made a quit attempt during this project, one had recently relapsed after quitting (without professional support), and one had never attempted to quit. Two parents were unemployed and perceived their income as insufficient, while the third parent reported her income as barely sufficient. Reported barriers to quitting smoking included mental health problems, financial stress, lack of motivation to quit, and a pro-smoking social environment.

**Table 1:** Characteristics of participants

Participant	Role	Gender	Income sufficiency	Reported vulnerability factors	Smoking status
Parent A	Mother of a 2-year-old child	Female	Insufficient (receiving social welfare benefits)	Receiving community care support; distance to labour market	Smokes ~ 14 cigarettes/day; no other tobacco or nicotine use; two previous quit attempts
Parent B	Mother of a 2-month-old infant	Female	Borderline	Anxiety disorder and depression; pro-smoking family environment	Smokes ~ 2 cigarettes/day; no other tobacco or nicotine use; recent relapse after short abstinence
Parent C	Mother of three children (0–8 years)	Female	Insufficient (under debt administration)	High stress; previous substance use disorders; pro-smoking social norms	Smokes ~ 14 cigarettes/day; no other tobacco or nicotine use; no previous quit attempts or intention to quit
Counsellor A	Smoking cessation counsellor	Female	Sufficient	Not applicable	Never smoked
Counsellor B	Smoking cessation counsellor	Female	Sufficient	Not applicable	Never smoked
Professional A	Youth health care nurse specialist	Female	Sufficient	Not applicable	Never smoked
Professional B	Midwife	Female	Sufficient	Not applicable	Never smoked
Researcher A	Researcher, paediatrician in training	Female	Sufficient	Not applicable	Previously smoked
Researcher B	Researcher	Female	Sufficient	Not applicable	Never smoked

### Ethical considerations

Ethical approval for this study was obtained from the Erasmus MC Non-WMO Review Committee (MEC-2023–0571). Participation in the co-creation sessions was classified as minimal risk. To safeguard participants’ confidentiality, a set of practical measures was implemented. Group rules (e.g., on confidentiality and respectful communication) were jointly established at the start. The sessions were held in an informal setting at the same location, with the same participants, to support trust and continuity. Researchers conducted short individual follow-up calls to ensure participants felt comfortable with their involvement [43]. Participants were reminded that they could pause or withdraw at any time. Their contributions were acknowledged with financial compensation (€25 per session), and travel expenses were reimbursed.

### Study design and process

This study adopted co-creation as a methodological approach within a participatory health research paradigm, in which service users, professionals, and researchers collaborate throughout the research process to inform intervention development [44–46]. In line with this approach, this study followed Leask et al.’s [24] five guiding principles and co-creation framework (PRODUCES). Table 2 details how the PRODUCES framework was applied to plan this study, following Principle 1 (Planning). Sampling procedures (Principle 2) are described above. During the first co-creation session, participants jointly framed the project aim (Principle 3: Manifesting ownership), were introduced to the existing SFP programme, and collaboratively defined group rules (Principle 4: Defining the procedure). The next four sessions, as outlined in Table 3, included interactive methods adapted from Leask et al. [24], the Co-creation Impact Compass [48], and Butterfly Works [47]. The Co-creation Impact Compass and Butterfly Works toolkits are freely accessible, practice-oriented guides that offer a broad range of structured participatory exercises, allowing researchers to select activities that best fit the aims and context of their co-creation project. Activities included photo-based brainstorming, peer interviews, creating fictional user profiles (‘persona’), user journey mapping, and traffic light prioritization. Fieldwork assignments were conducted between sessions to deepen insight and served as input for subsequent discussions [24, 49].

**Table 2:** PRODUCES study planning

PRoblem	There are substantial disparities in smoking prevalence and smoking cessation interventions often fail to effectively engage and support parents in vulnerable situations
Objective	To tailor Smoke-Free Parents to better engage and support parents in vulnerable situations
Design	We applied a Participatory Health Research approach, which emphasises collaboration with participants and the joint development of practice-oriented interventions [44–46]
Users	Parents who smoke
Co-creators	Parents in a vulnerable situation, healthcare professionals, smoking cessation counsellors, academic researchers
Evaluation	Process (interviews), content, and effect (pilot study) evaluation
Scalability	Generalizable model

**Table 3:** Content of the co-creation sessions

Session	Preparatory fieldwork	Session content	Applied methods
Individual intake (1 h)		Introduction to the co-creation methodology and Smoke-Free Parents; brief interview on personal background and experiences with smoking (cessation)	Individual semi-structured intake interview (to build rapport and understand participant context)
1: Introduction (2 h)		Getting to know each other; introducing Smoke-Free Parents; sharing motivations; (re-)framing the project aim; defining group rules; exploring associations with smoking and cessation through visual mind mapping	Visual mind mapping (participants select images to express feelings and beliefs about smoking and quitting) [47]
2: Understanding context and needs (2 h)	Making a collage (on a template) of the persona of a fictional user, its behaviour, emotions and thoughts, context and needs	Discussing fictional user profiles; identifying barriers and facilitators to accessing Smoke-Free Parents and quitting smoking	Persona collage [47, 48] (visualising lived contexts and needs of diverse users)
3: Understanding context and needs (2 h)	Informal interview with a (expectant) parent outside the group [47]	Building on previous session and interviews; identifying barriers and needs across stages of the user journey	Interviewing [47] and user journey mapping (visual mapping of barriers and needs across stages of the participant journey) [47, 48]
4: Brainstorming solutions (2 h)		Finalizing barriers and facilitators per user journey stage; subgroup brainstorming of potential solutions	Design Charette [48] (structured brainstorming exercise in subgroups, followed by plenary refinement)
5: Prioritizing and evaluating (2 h)		Prioritizing ideas and evaluating the co-creation process and results	Traffic light prioritization (based on MoSCoW method [48], participants individually classify adaptations as high [green], medium [amber], or low [red] priority to identify feasible and acceptable changes)

### Process evaluation

Process evaluation was conducted in line with Principle 5, covering three process criteria (validity, satisfaction and ownership) and two outcome criteria (participant satisfaction and effects) [24]. Validity was supported through member checking at the start of each session, where key insights were summarized and discussed with co-creators for refinement and verification. In addition, individual post-session interviews with all seven non-researcher participants and a group discussion in the final session were used to assess the perceived validity and appropriateness of the process and outcomes. To safeguard confidentiality during the group discussion, individual feedback was collected in writing without identifying information and did not require sharing within the group. Satisfaction was explored via follow-up telephone interviews after each session. Ownership was assessed through attendance and retention. The outcome evaluation was based on co-creators’ feedback on the developed intervention and the planned implementation of a pilot study (outside the scope of this article). Interview and group discussion guides used for evaluation are provided in Supplementary File 1 and were informed by the Community-Based Participatory Research model [50] and the InSPIRES Impact Evaluation framework [51]. Feedback from the process evaluation consisted of both quantitative ratings and qualitative comments. Data were analysed using a descriptive synthesis approach, in which ratings were summarised and open-ended feedback was grouped by the process evaluation criteria of validity, satisfaction and ownership.

### Content evaluation

This section describes the thematic analysis of the contexts, needs, and proposed adaptations identified during the co-creation sessions.. Audio records of the sessions were transcribed verbatim. Three researchers (LvdS, LJB and ALdB) familiarized themselves with the data by reviewing transcripts, meeting notes, and creative outputs, and summarizing key insights in a synthesis report discussed with co-authors (LvdS, LJB and TSvS) and stakeholders. We conducted a hybrid thematic analysis, combining deductive and inductive coding [52]. Deductive coding was guided by the Capability, Opportunity, and Motivation Behavioural model (COM-B) [53], which provided the main coding categories. Within each COM-B domain, inductive coding was used to identify more specific subthemes grounded in participants’ input and the real-world intervention context. Proposed programme adaptations were analysed within four predefined stages of the participant journey (1a. Awareness and self-registration; 1b. Referral; 2. Coaching; and 3. Aftercare), which also structured the data collection. Within each COM-B domain and journey stage, inductive coding was used to identify more specific subthemes grounded in participants’ input and the real-world intervention context. This process involved open, line-by-line coding. Coding was conducted independently by two researchers (LvdS and ALdB) using MAXQDA 2022. During regular analytic meetings interpretations were compared, discrepancies were discussed, and the coding framework was iteratively refined. A third researcher (LJB) was regularly involved in these discussions and in refining the coding framework. In subsequent analytic rounds, related codes were clustered into broader themes through team discussion, focusing on patterns that recurred across participants and sessions. An overview of all codes used in the analyses is available in Supplementary File 2.

## Results

This section presents the synthesised outcomes of the co-creation process, including contextual factors, needs, and proposed adaptations. Together, these outcomes illustrate how contextual circumstances informed participants’ proposals for adapting the Smoke-Free Parents programme.

### Context and needs

#### Capability

Participants described the physical difficulty of overcoming nicotine addiction and managing withdrawal symptoms as a major challenge. Alcohol use, mental or physical health problems, or addressing multiple addictions simultaneously further reduced perceived capability. For some, previous failed attempts to quit led to the belief that quitting is impossible, while for others these experiences increased willingness to seek external support:

Professional B: *“The reason why she would want to make use of Smoke-Free Parents is because she has already tried so many ways [to quit]. And it just doesn't work. So she hopes that there will be enough know-how from Smoke-free Parents, that this could actually help her."*

In addition, participants noted that many parents and professionals lack awareness of the availability and effectiveness of cessation services. Parents’ lack of knowledge on the health risks of smoking for themselves and their children and the paradoxical role of smoking in stress relief, further limited their inclination to seek help. To address these barriers, participants emphasised the need for accessible and personalized information about the health risks of smoking and the cessation support options available, including clarity on reimbursement and how programmes can be tailored to individual needs (e.g., also addressing diet, alcohol use, or stress) and preferred formats (e.g., group, telephone, or face-to-face). Practical guidance was seen as crucial, covering what to expect during the quit attempt, how to prepare, what tools or medications are available, and how to cope with withdrawal, cravings, stress, and relapse. Some parents valued learning specific coping strategies, such as breathing exercises, provided these were adapted to individual preferences and circumstances.

#### Opportunity

Participants described how quit attempts are complicated by stressful life circumstances, such as financial hardship, relationship issues, unstable housing, or disrupted routines. Easy access to cigarettes and pro-smoking norms in their social environments further undermined quit attempts. Shame and stigma, particularly around smoking while pregnant or parenting, discouraged participants from disclosing their smoking or seeking help. Healthcare professionals expressed similar hesitancy, fearing that addressing smoking might be perceived as repetitive or unwelcome.

Parent C:* “Yes, but precisely because of this taboo, as a mother I don’t go to any service and say, I’m pregnant and I want to quit.”*

Professional A:* “But because of this taboo, I also don’t want to bring it up at every consultation. Because then I also think, you’re probably thinking, here comes that nagging from the clinic again.”*

To overcome these barriers, parents and professionals highlighted the importance of a trusting relationship with a non-judgmental and empathetic professional who tailors support to individual circumstances. Key conditions identified included accessible, low-threshold services, rapid follow-up after registration, tailored materials, and flexible delivery options, such as evening or weekend sessions, group meetings and peer support. Clear communication about what to expect, including the identity of the counsellor, was considered essential. Participants emphasised the involvement of partners, family, and friends. When introduced by the researchers, CO monitoring and financial incentives were viewed as helpful additions to support progress, provided clear explanations were given and safeguards would prevent misuse. Continuity of care and longer support trajectories were seen as particularly important for parents facing multiple challenges. Free-of-charge support was considered crucial to enable initial and repeated quit attempts and to alleviate concerns about unforeseen costs. Counsellors reported difficulties including unreachable participants (e.g., due to missing contact details) and missed appointments.

#### Motivation

Participants described smoking as a coping mechanism for stress and as a deeply ingrained habit tied to daily routines and a wide range of emotional triggers, both negative (e.g., boredom, loneliness, tension) and positive (e.g., relaxation, enjoyment, togetherness). Factors reducing motivation to quit included fears of weight gain, illness, stress, shame, and social judgment. At the same time, parents mentioned strong motivators such as the rising cost of cigarettes and the desire to protect their children’s health.

*Parent C: “The only thing motivating me to quit right now is the price. Other than that, I really don’t care at all, but it’s just getting too expensive. (…) Not for my health, not for my children. I just think, it’s just 27 euros now. That’s just not doable.”*

Confidence in one’s ability to quit was considered essential, yet previous failed attempts often undermined this belief. Counsellors emphasised the importance of normalizing relapse, viewing it not as failure but as an opportunity to re-engage with support and learn from the experience. To sustain motivation, parents and professionals highlighted the need for ongoing encouragement throughout the quitting process. Recognition of progress and health benefits, regular check-ins, and feedback from professionals were seen as crucial in maintaining commitment and building self-efficacy. All participants agreed on the value of realistic success stories, including experiences of relapse, to normalize setbacks and show that long-term success remains achievable.

Parent B: “*Yes, it’s a bit tricky as a GP, but just checking in with, ‘Hey, are you still smoke-free, how’s it going?’ and then acknowledging your progress, giving a moment of recognition if you’re successful. I think that’s important too, because at first it’s all new and exciting, all the compliments and pats on the back. But over time that wears off a bit, so it can actually be nice to have someone check in again every now and then.”*

### Proposed adaptations to the programme

**Fig. 1:**
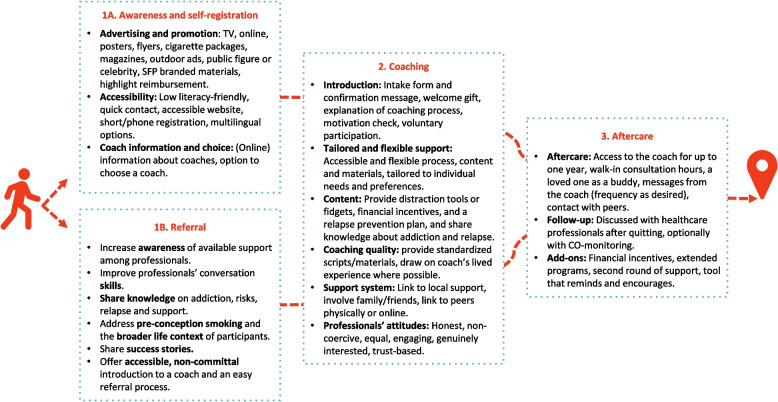
Proposed adaptations to the SFP programme. Adaptations were grouped by four stages of the participant journey. Participants could enter the programme either through self-enrolment (stage 1a) or referral by a healthcare professional (stage 1b)

Participants collaboratively developed adaptations aimed at improving the effectiveness and accessibility of SFP for (expectant) parents in vulnerable situations, in response to the contextual barriers and needs identified in earlier co-creation sessions. The proposed adaptations were developed and prioritised along the participant journey and are presented in Fig. 1.

For stage 1 (*enrolment and referral*), participants emphasised increasing awareness of support through diverse communication channels, simplifying registration, and enabling more personalised engagement such as choosing a counsellor. Healthcare professionals, as crucial referrers, were seen to require better knowledge of SFP, skills for supportive dialogue, and straightforward referral pathways.

For stage 2 (*coaching*), participants recommended a clearer intake process and flexible delivery options including online, in-person, group, or partner sessions. Parents valued flexible scheduling and involvement of local professionals who understand their context, while counsellors raised concerns about feasibility outside office hours and stressed the need for scheduling tools and standardised scripts to ensure consistency. Parents also supported culturally resonant materials, attention to stress or lifestyle topics, relapse prevention, social support, and carefully designed incentives. Counsellors suggested normalising relapse as a “slip-up”, while a parent expressed concern that this might lower the threshold for lapses.

For stage 3 (*aftercare*), participants highlighted the importance of continued support from counsellors, reimbursement for extended or repeat trajectories, routine follow-up by healthcare professionals, and if desired, peer or social network involvement.

### Evaluation of the co-creation process

This section summarises the authors’ synthesis of feedback provided by all non-researcher participants (*n* = 7) on the co-creation process. Findings are based on a descriptive synthesis of anonymous session ratings and open-ended feedback on satisfaction, ownership, and validity (see Supplementary File 1 for full evaluation data). Overall *satisfaction* was high: session ratings averaged 7.7–8.3 out of 10, with participants appreciating the respectful atmosphere, opportunities for exchange, and meaningful contribution. *Ownership* was strong, with no dropouts and an attendance rate of 94%. Participants valued the limited group size and clear communication and structure, though some suggested broader participant diversity (e.g., fathers, different age groups, pregnant women) and more efficient use of time. In terms of *validity*, most found the goals and procedures clear, but both a parent and a professional indicated that the project’s focus on tailoring for a specific group of parents could have been communicated more explicitly.

The co-created *outcomes* received positive evaluations (average 8.3/10), being described as rich and comprehensive. At the same time, participants highlighted the importance of ensuring follow-through and concrete impact: while many expressed optimism, they also noted uncertainty about actual implementation. The adapted intervention’s feasibility, reach, retention, satisfaction, and cessation *outcomes* are now being tested in a pilot study.

## Discussion

This study identified contextual factors and needs regarding smoking cessation support for parents in vulnerable situations and facilitated the collaborative proposal of adaptations to improve the SFP programme. Parents, smoking cessation counsellors, and healthcare professionals described how limited capability (e.g., physical dependence and lack of risk awareness and support), restricted opportunity (e.g., stressful life circumstances and limited social and professional support), and fluctuating motivation (e.g., stress relief, shame, concern for children’s health, and self-efficacy) constrained quit attempts and accessing quit support. These findings mirror prior evidence that socioeconomic disadvantage compounds the challenges of quitting [12, 31, 54, 55]. In this study, these contextual challenges were translated into practical insights into how stakeholders propose to address them, including via: (1) raising awareness of available support, (2) embedding trust, personalization, and flexibility in delivery, and (3) integrating social support, stress management, relapse prevention, and continuity of care. In line with the design-oriented nature of this study, the proposed adaptations are best interpreted as co-created hypotheses for programme improvement, rather than as empirically tested or universally applicable solutions.

Across discussions of contextual barriers to smoking cessation support, a recurring theme was the limited awareness of the availability, effectiveness and free-of-charge nature of smoking cessation support, among both professionals and parents. The persistence of this issue [7, 31] underscores participants’ call for clear communication, using personal and realistic stories to explain what coaching entails and why it could work. Consistent with prior evidence [55], participants stressed the need for empathetic, non-judgmental conversations. Training professionals in motivational interviewing, risk communication, stress-aware and trauma-sensitive approaches, and engaging participants with low literacy may help bridge this gap [12]. Trust and engagement were further linked to personalized counsellor matching (e.g., cultural background) and flexible delivery formats. Parents also valued lived experience, suggesting that support from peers or ex-smoker counsellors would help [56, 57]. Partners, family, and friends were sometimes described as barriers (through norms and triggers), as well as potentially impactful facilitators if given concrete guidance [54, 55]. Parents further highlighted the need for optional, tailored components such as stress management or lifestyle advice [12]. The intervention components CO monitoring and incentives were valued once explained, and participants expressed the importance of a supportive rather than controlling use of monitoring and safeguards against gaming in incentive schemes. These preconditions underline the importance of clear communication and co-design to foster acceptance [58]. Participants also emphasised extended aftercare, reimbursement for repeat attempts, and proactive check-ins, echoing guidelines that long-term continuity and relapse prevention are key components of effective cessation support [59, 60].

From a methodological perspective, this study illustrates both the strengths and limitations of using co-creation as an approach for intervention tailoring. The involvement of parents, counsellors, and healthcare professionals enabled the integration of experiential, professional, and scientific knowledge, supporting the development of context-sensitive and practically grounded proposals. At the same time, the transferability of the findings is limited by the small group size, the underrepresentation of fathers and partners, and a likely selection bias towards participants who were able and willing to engage in an intensive co-creation process. Conversely, the small but consistent group across multiple sessions, combined with ethical safeguards, fostered depth, continuity, and mutual trust, as reflected in participants’ process evaluation feedback. These features are widely recognized as core strengths of participatory research [25]. More broadly, co-creation can be seen as part of a broader iterative cycle of evidence-informed intervention development, in which participatory design is combined with existing research evidence and informs subsequent evaluation and implementation efforts [25]. This study also demonstrates how co-creation can surface tensions between stakeholder perspectives. Parents favoured flexible delivery, lived experience, and explicit risk communication, while professionals expressed concerns about the feasibility of these preferences and worried that discussing risks too explicitly might harm rapport with clients [12, 61]. Counsellors proposed framing relapse as ‘slip-ups’, while a mother worried this could lower the threshold for lapses. Such dialogues may support balanced decision-making and help identify conditions under which adaptations are both acceptable for parents and workable for providers [25].

Future studies should test the proposed adaptations in larger and more diverse populations, including fathers and partners, and in settings beyond the Netherlands, to strengthen external validity. In addition, research is needed on how co-creation can be embedded across routine cessation support, so that intervention design systematically incorporates the needs and perspectives of those who experience the greatest barriers.

## Conclusions

This study demonstrates that co-creation is feasible and valuable for tailoring smoking cessation programmes to the realities of parents in vulnerable situations. It yielded concrete adaptations now being piloted, including buddy support, aftercare, CO monitoring with feedback, and financial incentives. More broadly, it highlighted the value of co-creation as a democratic space where experiential, professional, and scientific knowledge are integrated. Embedding co-creation more widely in the development and adaptation of cessation support may support improvements in perceived accessibility and trust, thereby contributing to reducing persistent socioeconomic inequities in tobacco-related health outcomes.

## Supplementary Information

Supplementary Material 1.

Supplementary Material 2.

## Data Availability

Anonymized data may be shared upon reasonable request to the corresponding author.

## Abbreviations

COM-B: Capability, Opportunity, and Motivation Behavioural model
PRODUCES: PRoblem, Objective, Design, Users, Co-creators, Evaluation, and Scalability
SFP: Smoke-Free Parents
