# Supplementary material for: Tailoring a national smoking cessation support programme in co-creation with (expectant) parents in vulnerable situations
Source: BMC Public Health. 2026 Feb 14;26:945. doi: 10.1186/s12889-026-26633-9 (PMC13011541; doi:10.1186/s12889-026-26633-9)
Supplement: Supplementary file 1 — Supplementary Material 1. [file 12889_2026_26633_MOESM1_ESM.pdf]

Supplementary File 1: Process evaluation: topic lists and responses

Interim evaluations (individually)

Unstructured discussions by telephone covering roughly the following aspects after each session. Sometimes only the "top, tip, key insight, additions, and rating" were asked, via WhatsApp.

😁 Top: What was great?
☹️ Tip: What could be improved?
🤓 Learning: What did you learn?
😊 Comfort: Did you feel at ease? (Were others respectful towards you?)
👂 Communication: Did people listen to each other well?
🕐 Organization: What did you think of the organization? (Too slow/fast/disorganized/etc.?)
🏁 Goal: Was the goal of this project clear? Do you think it is achievable?
❓ Unclear: Were there things that remained unclear?
💭 Other: Is there anything else you would like to say?
⭐ Rating: What score would you give this session? (Based on the aspects just discussed, independent of the content)
⚠️ Key insight: What was the most important thing for you that was discussed during this session?
👉 Additions: Were there things not discussed that you consider important?

Summary of responses:

Session 1

- Vulnerability: "The only thing that struck me was whether we are designing this to really help the vulnerable group or not. And if we discuss it this way, how does that feel for the mothers who are present?"
- Sharing: Revisit the goals and share them in the WhatsApp group. Maybe also share the schedule for the upcoming evenings.
- More parents: "Will other people who smoke also join? Then I won't be the only one."
- One mother mentioned as her *top*: "That there were different people with different stories. You can learn a lot for yourself, while at the same time helping to improve the programme." She felt at ease and thought it was very pleasant. This applied to the other participants as well. Everyone rated the evening with an 8 or 9.
- Professional: "Great to agree on the ground rules together. It was presented positively." "The evening had plenty of humor, but they also checked whether the group was okay with that humor, which I appreciated."
- Average score: 8.3

Session 2

- Average score: 8.3
- Positive reactions. Good atmosphere, interesting insights.
- Give mothers even more opportunity to speak.

- A proper break would be nice.

Session 3

- Average score: 7.7 (four 8s and two 7s)
- Session was a bit messier, better time management needed, but content was again very interesting.
- Key points according to participants: involvement of family and friends in the quit attempt and/or the option of group contact; what the counsellor should do to support successfully; clear communication about what the programme entails; use of videos/images; lived experience; local coaching.

Session 4

- Average score: 8.3 (three 9s, two 7.5s, one 8)
- Enthusiasm about this session, well organized.
- One mother said: “We did not really go into how we can reach different layers of the population. And I think that is very important.”

Final evaluation (as a group)

The anonymous answers to the evaluation questions are shown below, with the picture choice between square brackets.

⚠️ 1. What is the most important result of this project for you?

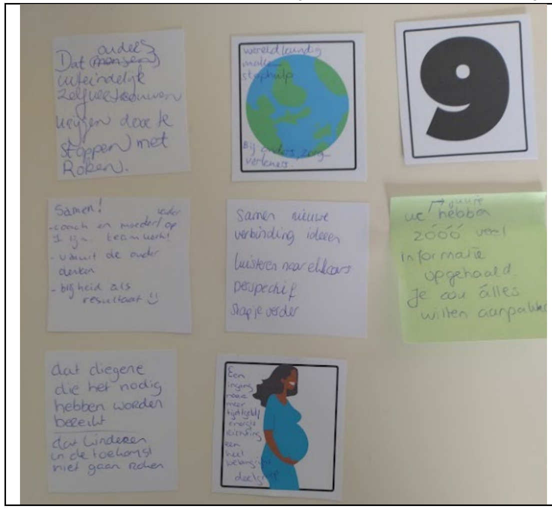

- We/you gathered sooo much information. You’d want to tackle everything. [Card with a number 9]
- A gateway to more time/money/energy for a very important target group. [Card with pregnant woman]
- That those who need it are reached. That children in the future will not start smoking. [Card with a girl]
- Together! 1) Counsellor and mother/father on the same page. Teamwork! 2) Thinking from the parent’s perspective. 3) Happiness as a result. [Card with people shoulder to shoulder]
- Together, new connections, ideas, listening to each other’s perspectives, taking a step forward. [Card with people shoulder to shoulder]
- Making smoking cessation support for parents known worldwide, to parents and healthcare professionals! [Card with globe]
- That parents ultimately gain self-confidence by quitting smoking. [Card with woman telling a story from a book]

🏁 2. Is the result of this project what you expected? Why or why not?

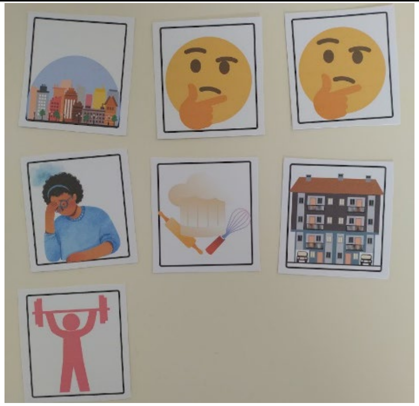	- The result is more than I expected. Smoking cessation care will become even stronger if improvements can be made / if it gains more visibility. [Card with weightlifter]<br>- No concrete expectations, but it was great to see that everyone had their own ideas, and valuable to learn from each other. [Card with baking utensils]<br>- Thinking together from different disciplines. I didn’t really have expectations beforehand. Nice how there was space for all ideas. [Card with thoughtful woman]<br>- It’s more enjoyable than I expected. Some really great ideas came out of it. Nice to go home with that each time. [Card with city]<br>- I didn’t know what to expect. [Thoughtful smiley]<br>- The first stones have been laid. Building further is a process. [Apartment building]<br>- I don’t really know, I don’t know what the actual outcome will be. But I did find the meetings encouraging. [Thoughtful smiley]

👉 3. What could we have done better?

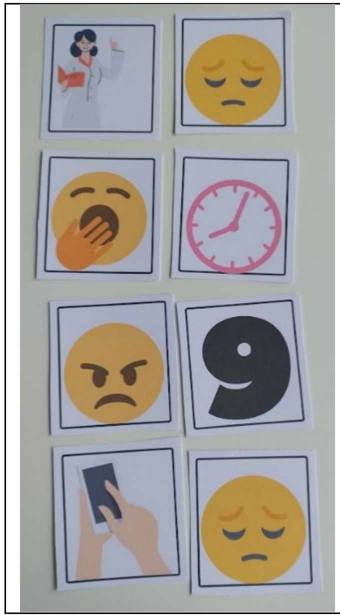	- It could have been more efficient, but maybe then we would have missed out on valuable conversations. Perhaps a bit more guidance to ensure equal input? [Woman speaking]<br>- Earlier in the evening (because of travel time, getting home late 😉) [Yawning emoji]<br>- That there is so little time to discuss everything. [Sad-looking emoji]<br>- More time. [Clock]<br>- Occasionally step in when the discussion strays too far from the topic. [Angry-looking emoji]<br>- A more diverse group of mothers, or perhaps more research into the psychological reasons why mothers continue smoking. [Number 9]<br>- That men (fathers) also join the group to talk about improvements. [Smartphone]<br>- Time should be monitored even more strictly. More parents (different ages, pregnant women, fathers) + counsellors + professionals. A larger group. Max. 12. [Sad-looking emoji]

⭐ 4. What grade would you give the final result?

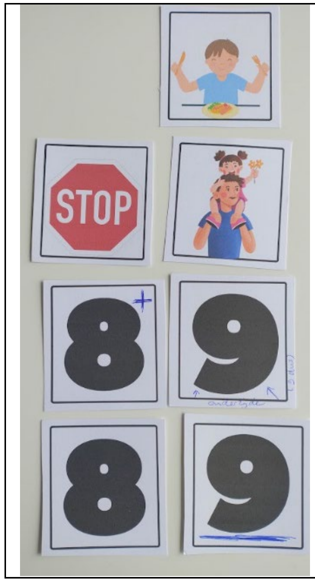	- 8! Curious to see what the “real” end result will be. What will be done with it? [Stop sign]<br>- An 8. I thought it went well. [Number 8]<br>- Lots of improvement tips in a pleasant way with nice people. [Number 9]<br>- [Number 9]<br>- 7 [Father with child]<br>- If there had been a larger group of parents, it would have been even more valuable. [Number 8+]<br>- I think an 8.5. [Person eating]<br>- **[Average score: 8.25]**

😊 5. What was the atmosphere like?

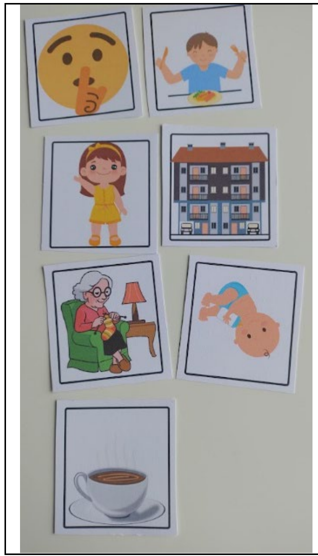	- Good, I was glad to be there. [Smiley with “silence” gesture]<br>- Nice to have dinner together! It was delicious, and the atmosphere was great! Open and honest. [Person eating]<br>- Pleasant atmosphere, you could say whatever you wanted. [Girl]<br>- I found it especially enjoyable, and the atmosphere was very open and trusting. [Apartment building]<br>- I enjoyed the pleasant atmosphere and appreciated being called afterward to share my thoughts. [Knitting grandma]<br>- Good, safe, and nice to hear from other people; it helps me look again with a fresh, curious, and open mind. [Baby]<br>- The atmosphere was great! A safe space to share everything. [Coffee]

6. Organization: How did you feel that everything was organized?

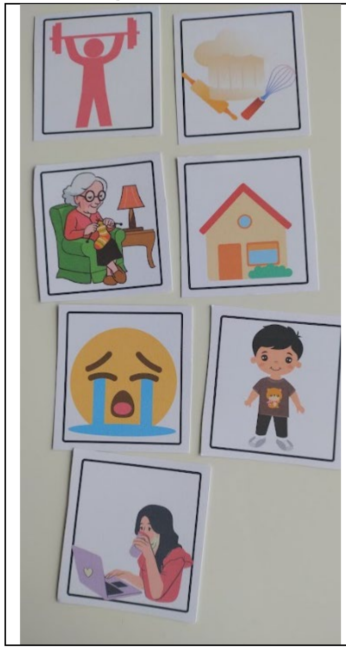	- 8. Everything was clear before, during, and after. Time management was more difficult (it was just too enjoyable). [Weightlifter]<br>- Fine. Drinks, snacks, break, location all good. [Baking utensils]<br>- In a pleasant way. Food/snacks/drinks. WhatsApp group. Well organized!! [Knitting grandma]<br>- Rock solid! Good communication before, during, and after the meetings. [House]<br>- Good group size. [Crying smiley]<br>- It was well organized. You felt at ease, got a follow-up call, and even received a little gift. [Boy]<br>- Organization: Fine, nice with food sometimes, recording was useful, slides/big screen handy, and nice to have a short introduction beforehand. [Woman behind laptop]

7. Goal: Was the aim of this project clear? What did you think of the aim?

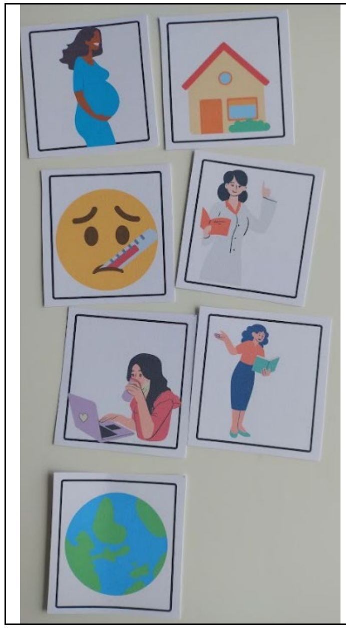

- Yes, clear! Quite general, but that was also the purpose 😉 [Pregnant woman]
- Yes, it was clear. Very good. Everyone should quit. [House]
- It was not clear to me that it was about a specific target group. [Sick smiley]
- For me it was clear. The aim was well defined. [Storytelling woman]
- I found the aim clear and also a particularly good one. Still, I wonder if we might have thought too generally. [Woman behind laptop]
- The initial aim was broad/vague. After the first session it became clearer together. Good to bring it back later on. [Storytelling woman]
- Not so clear beforehand. During the course it did become clear. [Globe]

💭 **8. Other: Is there anything else you would like to say?**

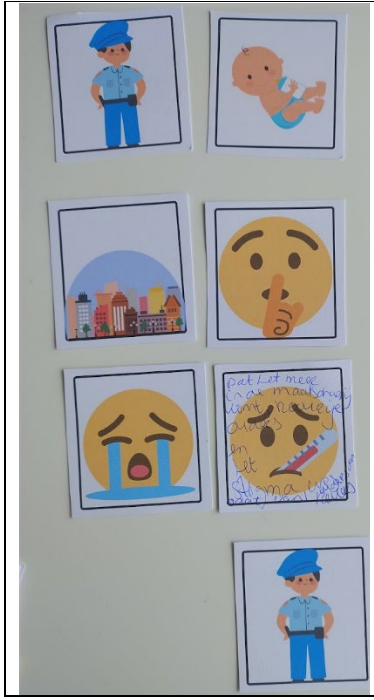	- Sometimes maybe cut off conversations that are not relevant. Even if they are fun 😊 [Policeman]<br>- I hope that the collected tips/info will really be reflected in practice. [Baby]<br>- I am very curious about the presentation and the reactions to it. I am quite proud to have contributed just a little bit to this co-creation. [Policeman]<br>- Best of luck with further development / bringing it to the attention of the organization / scaling up the intervention. [City]<br>- No. [Smiley with “silence” gesture]<br>- It’s nice that Smoke-free Parents is being improved and that it will continue to be, so it becomes better known. That it becomes more embedded in society, Smoke-free Parents. And that the stigma towards parents who smoke disappears. [Sick smiley]<br>- Too bad it’s over. [Crying smiley]

⭐ **9. What score do you give the co-creation method?**

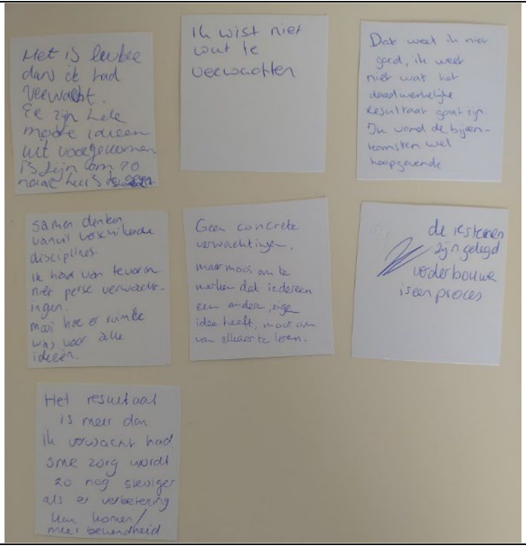

- 7. [Seven]
- 7.5 [Boy]
- Precisely the fact that multiple perspectives are included helps to improve the Smoke-free Parents programme. [7+1=8]
- 9. I really liked this way! Fun to think together like this. Hopefully you will have good results. [Four]
- Everyone has a say. Very nice. Sometimes a bit off-topic. At times I would have liked a little more steering to the point. But GREAT! [Ten]
- 9. [Crossed-out yawning smiley]
- 9. Nicely structured set-up. Everything was repeatedly summarized clearly in schemes. [One with a line through it]
- **[Average score: 8.25]**
