# Supplementary material for: Tailoring a national smoking cessation support programme in co-creation with (expectant) parents in vulnerable situations
Source: BMC Public Health. 2026 Feb 14;26:945. doi: 10.1186/s12889-026-26633-9 (PMC13011541; doi:10.1186/s12889-026-26633-9)
Supplement: Supplementary file 2 — Supplementary Material 2. [file 12889_2026_26633_MOESM2_ESM.pdf]

Supplementary File 2: Code book

The COM-B Framework was used for coding the first (context) and second theme (needs). COM-B means that Capability, Opportunity and Motivation are analysed as determinants of help-seeking and quitting Behaviour. Theme 3 explores proposed adaptations structured along the SFP participant journey.

Theme 1: Context and experiences of (expectant) parents who smoke

- ➔ Which factors in the individual's context determine whether they use cessation support and quit smoking?

Category	Code	Explanation	Example codes
**Capability** -Psychological: Knowledge & skills *(Does their mind allow them to change?)*	Knowledge about the effect of smoking on stress and relaxation	(Mis-)understanding of the relationship between smoking, stress, and relaxation.	"I didn't know that even a few cigarettes a day can be harmful."
	Knowledge about risks	Insight into the potential dangers and health consequences of smoking for the smoker and child.	
	Understanding how addiction works	Insight into the mechanisms of addiction and how this knowledge can contribute to quitting smoking.	
	Previous experiences with quitting	Experiences from earlier quit attempts, including what helped or hindered success, and how these experiences shape current motivation and confidence to quit.	
	Knowledge about available help	Insight into the available support and guidance for quitting smoking.	
	Mental health issues	Psychological or emotional difficulties such as anxiety, depression, or trauma that influence smoking behaviour or the ability to quit.	
**Capability** -Physical: Addiction (*Does their body allow them to change?)*	Nicotine addiction	Physical and psychological dependence on nicotine, influencing cravings, withdrawal symptoms, and difficulty quitting.	"I can't resist the craving for a cigarette."
	Health problems	Physical or mental conditions that can make quitting smoking more difficult or be experienced as a barrier.	
	Challenges in quitting multiple addictions simultaneously	Difficulties arising when trying to quit smoking alongside other addictive behaviours such as alcohol or drug use.	
	Influence of alcohol	Alcohol use affecting smoking patterns and cessation, including reducing self-control, increasing the likelihood of	

		relapse, and enhancing stress- and reward-related mechanisms that trigger smoking behaviour.	
**Opportunity -** Physical: Environmental factors (*How does their physical environment affect their opportunity to change?)*	Availability of cigarettes	Easy access to cigarettes in one's environment that undermines quit attempts.	"Help to quit is available, but I don't know where to start."
	Environment does not allow smoking	Situations and locations in which external circumstances or regulations make smoking physically or practically impossible, such as hospitalization.	
	Stressful living conditions	Situations that cause stress, such as financial issues, relationship problems, housing difficulties, or changes in life rhythm.	
	Availability of support	The extent to which smoking cessation support is accessible and offered.	
	Costs as a barrier to (second time) help	The financial barriers that make it difficult to seek smoking cessation support (again), such as limited or uncertain reimbursements and out-of-pocket costs.	
	Living in an area with low socioeconomic status (SES)	Growing up and/or living in an environment with lower average socioeconomic status.	
	Counsellor is unable to reach the parent	The counsellor is unable to reach the parent due to incorrect or missing contact details, unanswered calls or messages, or a lack of response.	
	Daily activities/work	A meaningful daily routine, creating structure, and increasing the sense of purpose and belonging.	
	Distraction from smoking	Activities that distract from smoking and reduce restlessness.	
	Opting for the most accessible support option	Choosing support that is quickly available, e.g., from the general practitioner.	
	Time constraints for healthcare professionals hindering discussions on smoking	Time constraints for healthcare professionals hinder them in discussing smoking and cessation.	
	Practical barriers towards participation in coaching	Factors that hinder participation in coaching, such as low literacy and competing priorities.	
**Opportunity -** Social (*How does their social*	Support from healthcare provider	Emotional and practical support provided by healthcare professionals to (expectant) parents in quitting smoking.	"My boyfriend also smokes, so quitting

environment affect their opportunity to change?)	- Shame/taboo hinders discussing smoking with healthcare provider<br>- Healthcare provider doesn't know when or how to start the conversation		feels even more difficult."
	Support from counsellor<br>- Open and honest conversation	Emotional and practical support provided by the counsellor to (expectant) parents.	
	Support from the environment<br>- Social support<br>- Influence of others' quitting in the social environment on one's own quitting<br>- Social network smokes, social pressure<br>- Cultural differences in smoking behaviour<br>- Smoking is socially accepted	Emotional and practical support provided by a person's social environment.	
**Motivation** -Automatic (emotions, habits)<br>*(Do their habits and emotions drive them towards change?)*	Habit formation<br>- Individual differences in smoking habits<br>- Grief and loss of habit	Smoking is an automatic behaviour, often linked to established routines, triggers, or situations, making it more difficult to quit.	"Smoking is my way of unwinding after a busy day."
	Coping<br>- With stress<br>- With boredom<br>- With social situations<br>- With mental health issues<br>- With relapse	Using smoking as a strategy or habitual response to certain situations or stimuli.	

	Guilt, shame, and taboo	Feelings of moral failure, stigma, or social disapproval associated with smoking, especially during pregnancy or parenthood.	
	Smoking gives positive feelings	Smoking provides positive feelings as it is associated with relaxation, contentment, and social connection	
**Motivation** -Reflective (intentions)<br>*(Do their beliefs and plans drive them towards change?)*	Motivation<br>- Motivation to stick with coaching<br>- Motivation to quit<br>- Quitting for freedom<br>- Quitting for yourself<br>- Quitting for your loved ones<br>- Quitting for your health<br>- Quitting for (future) child	The internal drive to quit smoking or to engage with cessation support, e.g., quitting for freedom means the person is driven by the wish to regain control and independence from addiction.	"I've tried so many times that I think I won't succeed."
	Self-confidence in ability to quit	Belief in one's own capability to quit smoking successfully.	
	Fear of weight gain, illness, stress, or addiction after quitting	Fear of weight gain, illness, stress, or taking up another addiction after quitting. Anticipated negative consequences of quitting may undermine motivation or confidence.	
	Recalcitrance<br>- Resistance to encouragement to quit from other (inexperienced) individuals	Consciously resisting the pressure to quit, possibly even smoking more.	
	Feelings of judgment<br>- Judgment from healthcare providers<br>- Judgment from social environment<br>- Judgment from counsellor<br>- Feeling of societal/social judgement (stigma)	Feelings of judgement about smoking, as an (expectant) parent, or in general.	

	Risk-reduction strategy	Behaviours or adjustments made by smokers to limit exposure to tobacco smoke for themselves or their environment, such as smoking only outside and considering the wind direction.	
	Self-reliance vs. support	The perception that quitting smoking is a personal responsibility, despite the availability of support.	
	Thinking that coaching doesn't help (enough)<br>- Thinking that coaching is too short	Thinking that coaching does not help enough to actually quit smoking, or that the coaching trajectory is too short for them to successfully and sustainably quit.	
	Smoking is expensive	The fact that smoking is expensive may serve as a motivation to quit.	
	Doubt/uncertainty about participation	Being in doubt about participation.	
	Doubt about health benefits and the value of quitting	The perception that quitting smoking offers little or no health benefits, based on personal or others' experiences, such as illness after quitting.	
	Fear of consequences of smoking registration	The concern that reporting smoking behaviour to a healthcare provider, followed by registration in electronic patient records, may lead to negative consequences, such as higher insurance premiums. This relates to trust in institutions and healthcare providers.	

Theme 2: Needs/preferences regarding smoking cessation support

➔ What do (expectant) parents in a vulnerable situation need to be able to use support and quit smoking?

**Categories**	**Codes**	**Definitions**	**Example codes**
**Capability** - Psychological: Knowledge & skills	Clear and practical guidance	The need for concrete, understandable, and structured information about smoking cessation support.	"It would be helpful if the healthcare provider openly

(What type of guidance or information do they need to feel capable of using support and quitting?)	Skills to cope with cravings	The strategies and practical actions someone uses to cope with cravings, such as exercising or drinking water. This includes both learned techniques and personal routines that help resist the urge to smoke.	talks about the risks of smoking."
	Knowledge sharing about…<br>- Coaching<br>- Addiction<br>- Reimbursement of support<br>- Health risks<br>- Smoking cessation<br>- Relapse<br>- Available support<br>- Other lifestyle factors	E.g., the need for clear and accessible information about what coaching entails, how the process works, and what participants can expect.	
**Capability -** Physical: Addiction (*What type of guidance or information do they need to feel capable of dealing with addiction of smoking?)*	Skills to manage breathing	Skills to effectively apply breathing techniques to manage stress, cravings, or psychological factors that may influence smoking cessation.	"I don't know what to do when I'm stressed."
	Skills to cope with addiction	Developing practical and psychological skills to manage cravings, withdrawal, and habitual smoking triggers.	
	Skills to cope with stress	Learning alternative strategies to handle stress and emotional tension without relying on smoking.	
**Opportunity** - Physical: Environmental factors<br>*(How should the support system be structured to be able to use support and quit smoking?)*	Getting an impression of the counsellor beforehand	The wish to see or read about the counsellor in advance to reduce uncertainty and increase trust and engagement.	"I need flexible support that fits into my schedule."
	Nicotine replacement products	The need for information about and access to nicotine replacement therapies or other pharmacological aids.	
	Financial incentives	Using monetary rewards or vouchers to support motivation and engagement in smoking cessation efforts.	

	(Funding for) extended coaching trajectories	The need for longer or more flexible coaching programmes that continue beyond the standard duration, supported by adequate reimbursement.	
	Financial reimbursement for (a second round of) support	Ensuring that repeat or renewed quit attempts can be reimbursed, preventing financial barriers to continued engagement.	
	Quick contact after registration	The expectation of prompt follow-up once registered for support to maintain motivation and reduce drop-out.	
	Appropriate coaching	Coaching that is tailored to the individual's circumstances, preferences, and readiness to quit.	
	Referral options for healthcare professionals	The need for clear, simple, and low-threshold pathways for professionals to refer parents to cessation support.	
	Easily accessible support	Availability of low-barrier, flexible support options (e.g., telephone, online, face-to-face) suited to users' practical and digital skills.	
**Opportunity** - Social<br>*(What kind of social support do they need to be able to use support and quit smoking?)*	Attitude of healthcare professional/counsellor<br>- Not feeling judged<br>- Trust-based relationship<br>- Coercive attitude of healthcare professional/counsellor<br>- Equal and respectful attitude	The interpersonal approach of professionals, which can strongly influence engagement, trust, and perceived safety.	"Talking to others who are in the same situation helps."
	Support from healthcare professionals<br>- The professional as a role model<br>- The professional has conversational skills<br>- Use of a CO monitor	Emotional and practical support provided by healthcare professionals to (expectant) parents in quitting smoking.	

	- Involvement of other healthcare professionals during/after quitting		
	Support from the counsellor<br>- Knowledge of the counsellor<br>- Skills of the counsellor<br>o Ability to deal with cultural differences<br>o Tailoring to the participant<br>- Referral to care for other problems<br>- Choice between phone call, video call or in-person session<br>- Counsellor has prior knowledge about the participant<br>- Support from the counsellor after the programme<br>- Counsellor from one's own community	Emotional and practical support during the SFP provided by counsellors.	
	Support from close relatives/loved ones	Emotional and practical support provided by the participants' social circle.	
	Lived experience of healthcare professional/counsellor	The lived experience of a healthcare professional or counsellor is valued, as it may increase connection and expertise.	
	Meeting peers with lived experience	Meeting peers with lived experience provides valuable support.	
**Motivation:** Automatic (emotions, habits) (*What kind of support do they need to feel encouraged and motivated and change habits?)*			"I want tips for dealing with stress."

**Motivation:** Reflective (intentions)<br>*(What features of support would make them more committed and motivated to use support and quit?)*	Need for motivation and encouragement	The desire for ongoing positive reinforcement and reminders of progress to maintain motivation throughout the quitting process.	"I need reminders and encouragement to keep going."
	Boosting self-confidence	Strengthening belief in one's own ability to quit smoking successfully, for example through feedback, praise, and achievable goals.	
	Sharing success stories	Valuing real-life, relatable examples of others' quit experiences (including relapses) to inspire, normalize setbacks, and sustain hope.	

Theme 3: Proposed adaptations

➔ **What adaptations can improve the effectiveness and accessibility of SFP for (expectant) parents in a vulnerable situation?**

General participant journey

**How can it be improved?**
➔ Attitude of counsellor/healthcare professional/parent<br>- Honesty<br>- Coercive attitude<br>- Equality<br>- Engagement and genuine interest<br>- Trust-based relationship

Station 1a: (Before) self-enrolment

**How can it be improved?**
➔ Awareness of support among end-users<br>- Advertising<br>○ TV commercials<br>○ Online advertisements<br>○ Posters & flyers (in waiting rooms)<br>○ Tobacco packaging<br>○ Magazines (e.g., *Ouders van Nu*)

  - “Nine Months” fair (*Negen Maanden Beurs*)
  - Outdoor advertising
  - Adding info to maternity packages
  - Promotion by a celebrity
  - Promotional materials (e.g., fidgets)
  - Awareness of support reimbursement
- ➔ Improving accessibility
  - For people with low literacy
  - Quick contact after registration
  - Accessible website for registration
  - Shorter registration form for participants
  - Telephone registration option
  - For non-native speakers
- ➔ Information about counsellors available
- ➔ Option to choose a counsellor

Station 1b: Conversation about smoking with a healthcare professional

**How can it be improved?**
➔ Awareness of support among healthcare professionals<br>➔ Knowledge sharing about risks<br>➔ Knowledge sharing about how addiction works<br>➔ Knowledge sharing about coaching<br>➔ Healthcare professional has conversational skills<br>➔ Healthcare professional must provide support<br>➔ Healthcare professional must address relapse<br>➔ Healthcare professional provides information about available support<br>➔ Healthcare professional must discuss smoking during the pre-conception phase<br>➔ Sharing success stories<br>➔ Non-committal availability of the counsellor<br>➔ Attention to the broader context of the participant<br>➔ Easy registration process by healthcare professional

Station 2: Coaching

How can it be improved?

- ➔ Introduction
  - Intake form
  - Confirmation email/message
  - Intake staff member
  - Knowledge sharing about what the coaching process looks like
  - Freedom to choose whether to participate or not
  - Not starting immediately, but keeping in contact with the counsellor at a later stage
  - Discussing motivation to quit
  - Welcome gift
- ➔ Tailored coaching
  - Choice between phone, video call, or in-person sessions
  - Ability to be coached together with a smoking partner
  - Customizable frequency of sessions
  - Possibility to extend the coaching programme
  - Relaxation exercises if desired
  - Attention to other lifestyle factors if desired
  - Option to change counsellor
  - Counsellor of similar age (life experience)
  - Counsellor from the same community
    - Appropriate materials (less perfection, more suited for men)
      - Digital materials (interactive)
      - Magazine in physical form instead of digital
      - For people with low literacy
- ➔ Use of distraction tools (fidgets)
- ➔ Relapse prevention plan
- ➔ Easier to schedule sessions (flexible, digital)
- ➔ Option to contact the counsellor via text or call outside regular sessions
- ➔ Scripts/standardized materials for counsellors
- ➔ Skills of the counsellor
  - Handling cultural differences
  - Tailoring to the participant

- ➔ Lived experience of the counsellor
- ➔ Counsellor in contact with other (local) healthcare providers and counsellors
- ➔ Financial incentives
  - Preventing misuse
  - Reward for keeping appointments
- ➔ Involving loved ones in coaching
- ➔ Knowledge sharing
  - Knowledge sharing about how addiction works
  - Knowledge sharing about relapse/slips
- ➔ Meeting peers with lived experience
  - Group programmes
  - WhatsApp/Facebook groups
  - Quit smoking app

Station 3: After coaching

How can it be improved?
➔ Aftercare (up to one year)<br>- Walk-in consultation hours<br>- A loved one as a buddy<br>- Messages from the counsellor (frequency as desired)<br>- Access to the counsellor<br>- Contact with peers with lived experience<br>➔ Follow-up by healthcare professionals after quitting<br>- CO monitoring<br>➔ Financial reimbursement<br>- Of extended programmes<br>- Of a second round of support<br>➔ Tool or fidget that reminds and encourages
